# Isolated, Closed Superficial Femoral Artery Rupture without Fracture Following Blunt Trauma; A Case Report and Literature Review

**DOI:** 10.30476/BEAT.2020.46454

**Published:** 2020-04

**Authors:** Deepak Kumar, Praveen Sodavarapu

**Affiliations:** 1 *Department of Orthopaedics, Post-Graduate Institute of Medical Education and Research, Sector 12, Chandigarh 160012, India*

**Keywords:** Blunt trauma, Femoral artery, Rupture

## Abstract

Injury to the femoral artery usually occurs either in open penetrating injuries or in association with fractures, but is unlikely with closed blunt trauma without fracture. We reported a 24-year-old female with a right-sided closed complete rupture of the superficial femoral artery without any bone injury and contralateral femoral shaft fracture following riding a bike and hitting by a tractor over both lower limbs. The right thigh and knee were swollen and tender with absent distal pulses without any knee instability. The left lower limb was shorter with crepitus and abnormal movement in the left thigh and intact distal pulses. Radiographs showed left femoral shaft fracture and no bony injury on the right lower limb. Angiogram showed non-opacification of the right distal superficial femoral artery. Fogartisation of distal and proximal ends were done and femoral artery was reconstructed using reversed saphenous vein interposition graft. So the clinical necessity of looking routinely for any arterial injury, even in cases of blunt trauma without bony injury is of great importance.

## Introduction

Most of injuries to the femoral artery occur either in open penetrating injuries or in association with fractures [1]. However, hemorrhage and pseudoaneurysm of deep femoral artery and pseudoaneurysm of superficial femoral artery due to blunt trauma without femur fracture have been reported in the literature [2-6]. Here, we reported a young female with a right-sided closed complete rupture of the superficial femoral artery without bony injury and contralateral femoral shaft fracture following run over by a vehicle.

## Case Report

**Fig. 1:**
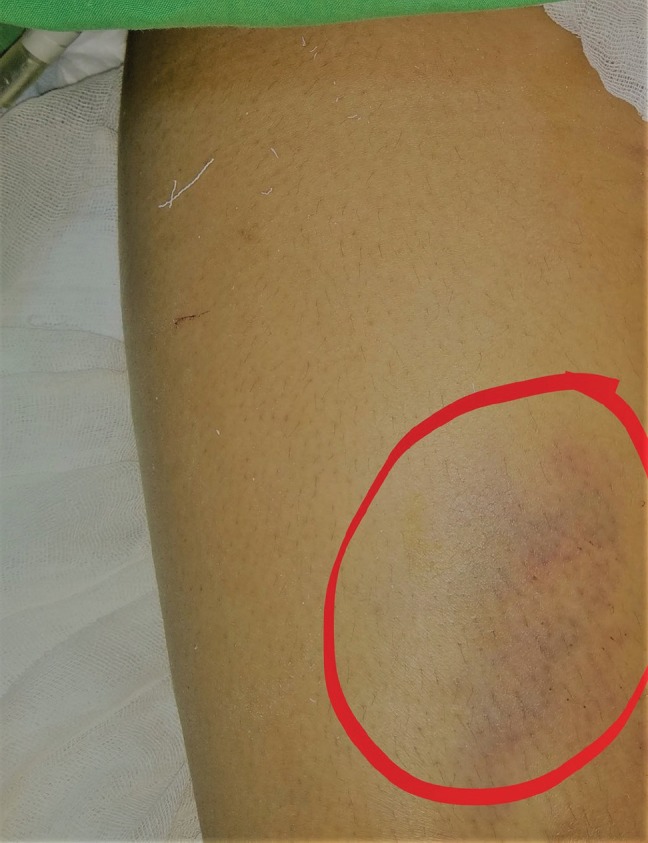
Marks on the skin indicating run over injury (encircled)

**Fig. 2:**
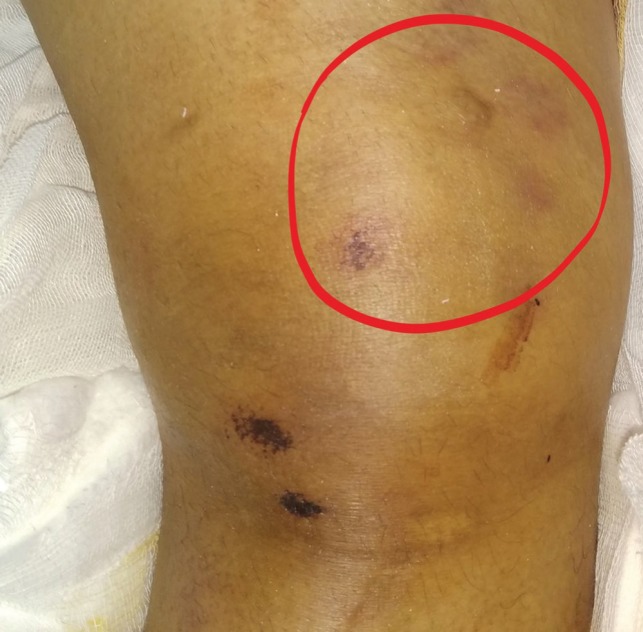
Marks on the skin indicating run over injury (encircled)

**Fig. 3:**
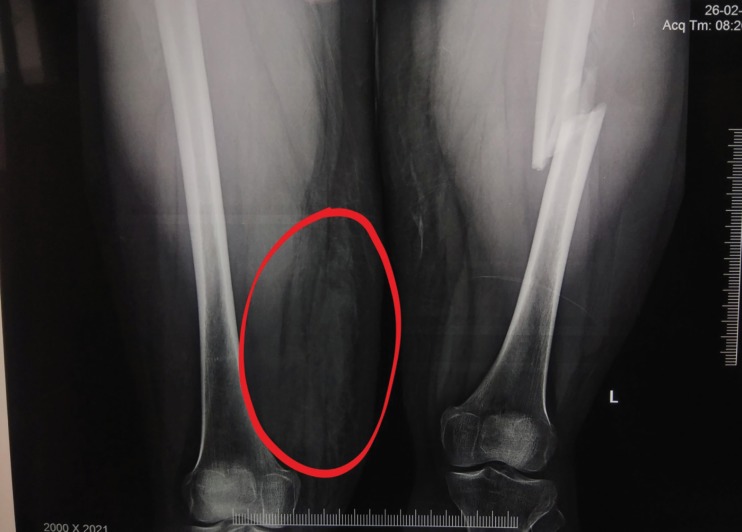
Radiographs showing left femoral shaft fracture and abnormal soft tissue shadow in the medial aspect of the right distal thigh (encircled)

**Fig. 4:**
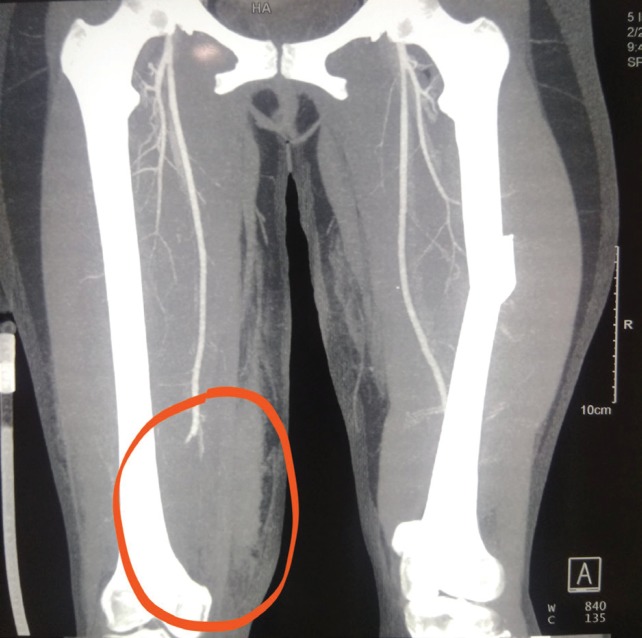
CT angiogram showing non-opacification of right distal superficial femoral artery (encircled)

A 24-year-old female sustained an injury, while pillion riding a bike, and she was hit and run over by a tractor over both lower limbs and was brought to the trauma center 5 hours after the injury. The patient was normotensive with a blood pressure of 110/80 mmHg and a pulse of 90/minute in the emergency department. The right thigh and knee were swollen and tender with absent anterior tibial artery (ATA) and posterior tibial artery (PTA) without any knee instability (Figure 1 and 2). Capillary refill time and pinprick were delayed. The limb was cold, associated with diminished toe and ankle movements, and diminished sensation over the foot. The left lower limb was shorter with crepitus and abnormal movement in the left thigh and intact distal pulses. Radiographs were performed, which showed left femoral shaft fracture and no bony injury on the right lower limb. Radiographs also showed abnormal soft tissue shadow in the medial aspect of the right distal thigh (Figure 3). Doppler showed absent ATA and PTA on the right side following which computed tomography (CT) angiogram was performed, which showed non-opacification of right distal superficial femoral artery (SFA) (length of 6 cm) (Figure 4) with popliteal artery reformation, non-opacification of ATA and opacification of PTA and peroneal artery. 

The patient was taken up for surgery, and the SFA was explored, which was found to be transected with a 7 cm contused segment of the vessel around 10 cm proximal to the knee joint. The contused segment was resected, fogartisation of distal and proximal ends was done, and reconstruction with reversed saphenous vein interposition graft was undertaken using 5-0 prolene. Medial and lateral fasciotomy for right leg was conducted and external fixator was applied for left femur fracture. 

**Fig. 5:**
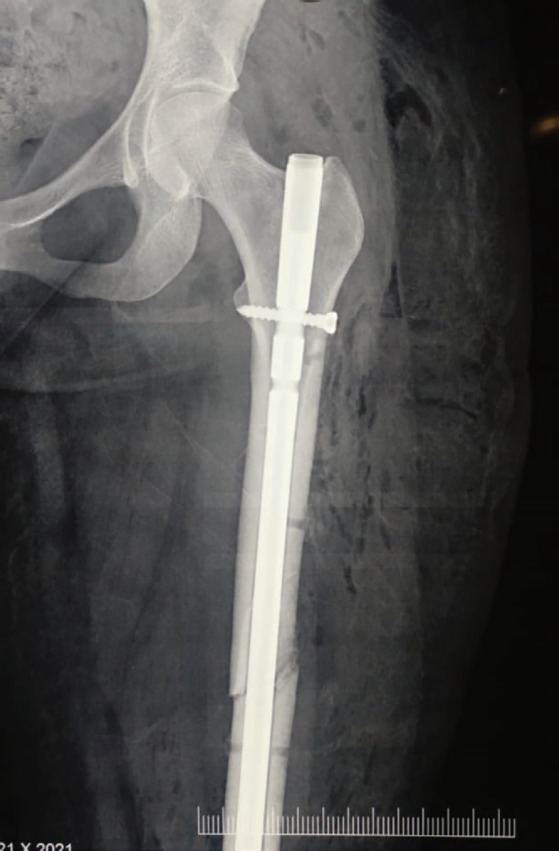
Closed reduction and internal fixation of left femur fracture using an intramedullary nail

Postoperatively, heparin and low-dose aspirin were given. The limb was warm with normal capillary refill, and Doppler showed biphasic flow in ATA and PTA. Fasciotomy wound was treated with vacuum-assisted closure and removal of the fixator and internal fixation using closed intramedullary nail (Figure 5), and primary closure of fasciotomy wound was performed after two weeks. Follow up of patient after one month was uneventful with palpable clinical pulses and normal sensation and movement in her right lower limb.

## Discussion

Injury to the femoral artery, although not frequent, is seen in association with open injuries, penetrating injuries, and concomitant bony injury. Isolated femoral artery injury without bony injury is less common following blunt closed trauma. Injury to the common femoral artery without pelvic or femur fracture has been reported in few instances, referred to as the motor-scooter handlebar syndrome, which was first described in 1968, and has been hypothesized to result from compression of the artery by the inguinal ligament [7]. 

Complete common femoral artery transection caused by a direct bicycle handlebar trauma requiring an emergency intervention and a bypass reconstruction has also been reported [8, 9]. Very few reports exist of isolated closed injury occurring after the division of the common femoral artery. Blasier and Pape reported rupture of branches of the deep femoral artery before [2] and pseudoaneurysm of the deep femoral artery without bone injury, which was ligated and excised was previously shown [3, 10, 11]. 

Superficial femoral artery injuries following closed blunt trauma without bone injury have been summarized in the table. Our case of isolated closed complete rupture of the superficial femoral artery without bone injury is unique as this type of injury has not been reported previously in the literature and has been reported for the first time, to the best of our knowledge. We hypothesized that the relatively fixed femoral artery, where it passes through Hunter's canal and close proximity to the bone makes superficial femoral artery vulnerable to blunt trauma, as in our case. 

The possibility of superficial femoral artery injury should also be considered in patients with closed blunt trauma and without bone injury. The primary care providers should be vigilant enough to make a note of telltale signs of soft tissue injury both clinically and radiologically, especially in patients with run-over injuries. This illustrates the clinical necessity of looking routinely for any arterial injury, even in cases of blunt trauma without bone injury.

**Table 1:** Blunt closed trauma causing injury to superfical femoral artery without bony injury

**Author**	**Mechanism of trauma**	**Duration of presentation**	**Injury to SFA**	**Procedure**	**Outcome**	**Contributing factor**	**Associated injuries**
Norris et al., [4]	Hit by basketball	6 months	Pseudo-aneurysm	Direct repair	-	Femur exostosis	-
Ramakantan et al., [5]	Hit by cricketball	2 months	Pseudo-aneurysm	Steel coil embolisation	No recurrence	-	-
Davis et al., [6]	Vehicle collison	1 month	Pseudo-aneurysm	Usg guided thrombin injection	No recurrence	Diabetes and hypertension	Ribs#, Ankle#, Sternal#
Angiletta et al., [9]	Fell on water tap	3 Hrs	Occlusion	Endovascular stenting	Viable limb	-	-
Our case	Run-over by vehicle	5 Hrs	Rupture	Reverse saphenous vein graft	Viable limb	-	Left Femur#

SAF: Superficial femoral artery

**Figures**

## Conflict of Interest:

The authors declare that there is no conflict of Interest.
